# RINCK-mediated monoubiquitination of cGAS promotes antiviral innate immune responses

**DOI:** 10.1186/s13578-018-0233-3

**Published:** 2018-05-09

**Authors:** Zhao-Shan Liu, Zi-Yu Zhang, Hong Cai, Ming Zhao, Jie Mao, Jiang Dai, Tian Xia, Xue-Min Zhang, Tao Li

**Affiliations:** 1 State Key Laboratory of Proteomics, Institute of Basic Medical Sciences, National Center of Biomedical Analysis, 27 Tai-Ping Rd., Beijing, 100850 China; 2 State Key Laboratory of Toxicology and Medical Countermeasures, Beijing Institute of Pharmacology and Toxicology, National Center of Biomedical Analysis, 27 Tai-Ping Road, Beijing, 100850 China

**Keywords:** RINCK, Monoubiquitination, cGAS, Innate immunity, Antiviral immunity

## Abstract

**Background:**

As an important danger signal, the presence of DNA in cytoplasm triggers potent immune responses. Cyclic GMP-AMP synthase (cGAS) is a recently characterized key sensor for cytoplasmic DNA. The engagement of cGAS with DNA leads to the synthesis of a second messenger, cyclic GMP-AMP (cGAMP), which binds and activates the downstream adaptor protein STING to promote type I interferon production. Although cGAS has been shown to play a pivotal role in innate immunity, the exact regulation of cGAS activation is not fully understood.

**Results:**

We report that an E3 ubiquitin ligase, RING finger protein that interacts with C kinase (RINCK, also known as tripartite motif protein 41, TRIM41), is critical for cGAS activation by mediating the monoubiquitination of cGAS. Using CRISPR/Cas9, we generated RINCK-deletion cells and showed that the deficiency of RINCK resulted in dampened interferon production in response to cytosolic DNA. Consistently, the RINCK-deletion cells also exhibited insufficient interferon production upon herpes simplex virus 1, a DNA virus, infection. As a result, the viral load in RINCK-deficient cells was significantly higher than that in wild-type cells. We also found that RINCK deficiency inhibited the up-stream signaling of DNA-triggered interferon production pathway, which was reflected by the phosphorylation of the TANK-binding kinase 1 and the interferon regulatory factor 3. Interestingly, we found that RINCK binds to cGAS and promotes the monoubiquitination of cGAS, thereby positively regulating the cGAS-mediated cGAMP synthesis.

**Conclusions:**

Our study reveals that monoubiquitination is an important regulation for cGAS activation and uncovers a critical role of RINCK in the cGAS-mediated innate immunity.

## Background

The innate immune system protects host from infections by detecting microbials and eliciting immediate immune responses [1]. The pattern-recognition receptors (PRRs) of the host are responsible for the effective recognition of danger signals from microbial [2, 3]. These danger signals, such as nucleic acids and toxins, are collectively called pathogen-associated molecular patterns (PAMPs). Although the cells evolved different kinds of PRRs for different PAMPs, the detection of nucleic acids provides the most efficient mechanism to sense invade microbials [2, 4].

Normally the DNA of eukaryotic cells is restricted in the nucleus or the DNA-containing organelle, mitochondrion. Upon infections, DNA of microbials will present in the cytoplasm and can be quickly detected by the host [4]. Recent study identifies cGAS as a key sensor for cytosolic DNA [5, 6]. When engaged with DNA, cGAS is activated and catalyzes the synthesis of cGAMP, which is a second messenger molecule that binds and activates stimulator of interferon genes (STING) [7–9]. The activated STING then recruits TBK1, which further activates the downstream transcription factors, IRF3 and NF-κB, to initiate the production of type I interferons and other inflammatory cytokines [10].

Since cGAS is a key sensor of cytosolic DNA in many cell types, the activation of cGAS must be tightly controlled. The dysregulation of cGAS activation has been implicated in several autoimmune diseases [11]. For example, cells are constantly exposed to genomic stresses, which give rise to the emergence of DNA fragments in the cytoplasm [12]. DNases, such as DNA Three prime Repair Exonuclease 1 (TREX1), are responsible for the degradation of cytosolic DNAs to prevent the autoimmune responses [13–15]. The deletion of *Trex1* in mice resulted in lethal autoimmune conditions [14]. Importantly, when crossed with cGAS knock-out mice, the TREX1-deficiency-induced lethality of *Trex1*^−*/*−^ mice is fully rescued [16]. This indicates that the uncontrolled cGAS activation is the major cause of TREX1-deficiency-induced death. Moreover, the aberrant activation of cGAS is also linked to other autoimmune diseases and aging [17, 18]. Thus, understand the regulation of cGAS activity is important for designing strategies to treat the cGAS-related diseases.

It has been reported that autophagy plays a critical role in regulating cGAS activation [19]. Post-translational modifications have also been shown to be important for cGAS regulation. For instance, Akt (also known as protein kinase B)-mediated cGAS phosphorylation inhibits cGAS activation in the late phase of DNA treatment and thus contributes to turning off the signaling [20]. Glutamylation has been reported to be an important regulation of cGAS [21]. Protein ubiquitination is a key regulatory modification in a variety of biological processes, including innate immune responses [22]. Ubiquitin is a highly conserved 76-amino-acid protein, and the addition of ubiquitin on a substrate protein is called ubiquitination. Protein can be modified by either a single ubiquitin (monoubiquitination) or a ubiquitin chain (polyubiquitination). An ubiquitin contains seven lysine (K) residues (K6, K11, K27, K29, K33, K48 and K63) [23]. When the polyubiquitination chain forms, the secondary ubiquitin is always linked to one of the seven lysine residues or the N-terminal methionine (M) of the previous ubiquitin. Similar to ubiquitination on other proteins, the K48-linked polyubiquitination initiates the degradation of cGAS, and the K27-linked polyubiquitination of cGAS mediated by the E3 ubiquitin ligase RNF185 promotes the enzymatic activity of cGAS [24, 25]. In this study, we found that E3 ubiquitin ligase RINCK plays an important role in cytosolic DNA and DNA virus-induced immune responses. Silencing of RINCK attenuated cytosolic DNA-triggered interferon production. Further, RINCK positively regulates cGAMP synthesis by mediating the monoubiquitination of cGAS. Our findings revealed that monoubiquitination is an important regulation for cGAS activation and uncovers a critical role of RINCK in the cGAS-mediated innate immunity.

## Results

### RINCK is required for cytosolic DNA-induced type I interferon production

To understand the detailed regulation of cGAS activation, we analyzed the interacting proteins of cGAS with a liquid chromatography-mass spectrometry (LC–MS/MS) approach. To do so, we expressed Flag-tagged human cGAS in cells and cGAS was immunoprecipitated with anti-Flag agarose beads. The co-precipitated proteins were separated by SDS-PAGE and further analyzed with LC–MS/MS. Among the identified proteins we selected several candidates and focused on RINCK in this study.

**Fig. 1:**
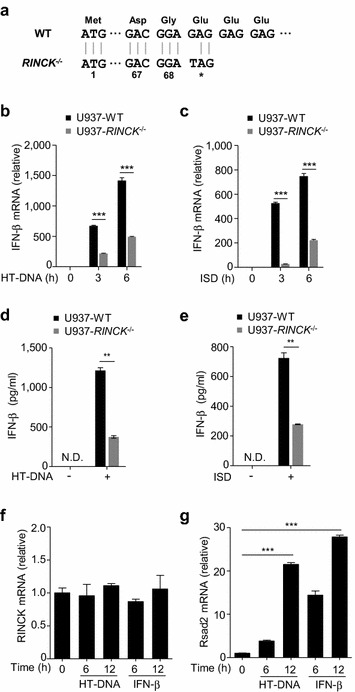
RINCK is required for cytosolic DNA-induced type I interferon production. **a** Schematic drawing of the RINCK deletion in U937 cells. **b**, **c** WT or RINCK-deficient U937 cells were treated with HT-DNA or ISD for indicated time followed by measuring interferon (IFN)-β mRNA with qPCR. **d**, **e** WT or RINCK-deficient U937 cells were treated with HT-DNA or ISD for 12 h, and the culture medium was collected for quantification of IFN-β by ELISA. **f**, **g** U937 cells were treated with HT-DNA or IFN-β for indicated time followed by measuring mRNA levels of RINCK and Rsad2 with qPCR. Data are presented as the mean ± SD. **P < 0.01, ***P < 0.001. N.D., not detected. Data represent three independent experiments

Since cGAS is a key DNA sensor, we first examined whether RINCK is involved in DNA-induced Type I interferon produce. Using clustered regularly interspaced short palindromic repeats (CRISPR)/Cas9 technology, we generated RINCK-deficient U937 (human monocytic cell) cells (Fig. 1a). We challenged the RINCK-deficient cells by introducing herring testis DNA (HT-DNA) into the cytoplasm and then detected the interferon production by quantitative PCR (qPCR) (Fig. 1b). We found that the interferon production level was significantly inhibited in RINCK knockout cells, compared to that of wild type (WT) cells. Consistent results were obtained when the cells were treated with interferon stimulatory DNA (ISD) (Fig. 1c). We further used enzyme-linked immunosorbent assay (ELISA) to measure the secreted interferon-β, our data show that the deletion of RINCK resulted in the dampened interferon-β synthesis in response to cytoplasmic DNA treatment (Fig. 1d, e). Thus, RINCK is critical for the type I interferon production induced by cytosolic DNA. We next examined whether the expression of RINCK mRNA is inducible in response to HT-DNA or interferon-β treatment. Our results showed that the expression of RINCK was not induced by interferon or cytosolic DNA treatment, while a known ISG, *Rsad2*, was dramatically induced (Fig. 1f, g).

### RINCK deficiency attenuates cytosolic DNA-triggered cGAS/STING signaling

**Fig. 2:**
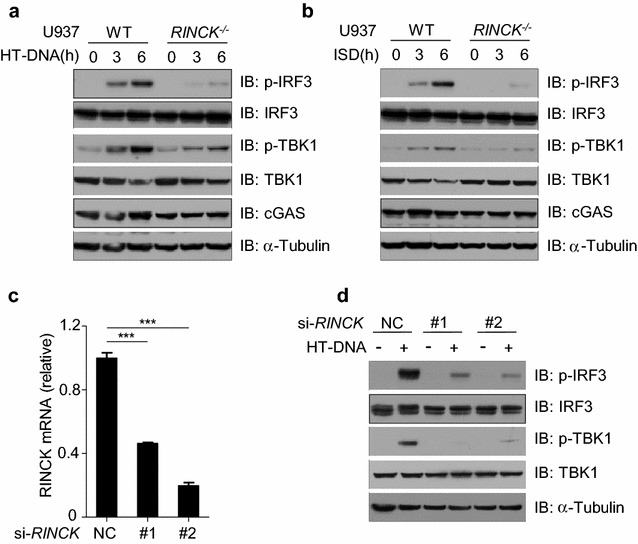
RINCK deficiency attenuates cytosolic DNA-triggered cGAS/STING signaling. **a**, **b** WT or RINCK-deficient U937 cells were treated with HT-DNA or ISD for indicated time followed by immunoblotting with indicated antibodies. **c** HeLa cells were transfected with the negative control (NC) or RINCK siRNAs for 48 h followed by measuring RINCK mRNA with qPCR. **d** HeLa cells were transfected with the negative control (NC) or RINCK siRNA for 48 h and then were treated with HT-DNA for 3 h followed by immunoblotting with indicated antibodies. Data are presented as the mean ± SD. ***P < 0.001. Data represent three independent experiments

To determine the role of RINCK in the DNA-triggered signaling pathway, we detected the activation of cGAS/STING signaling, which can be reflected by the phosphorylation of IRF3 and its kinase, TBK1. In line with our above findings, the deletion of *RINCK* led to significantly inhibited activation of IRF3 and TBK1 (Fig. 2a, b). To further confirm these observations, we used HeLa cells to study the role of RINCK in the cytosolic DNA-induced cGAS/STING signaling, as HeLa cells were previous used to study cGAS function [19]. We treated HeLa cells with siRNAs targeting RINCK or the control siRNAs for 48 h to efficiently knockdown the expression of RINCK (Fig. 2c). We found that knockdown of RINCK markedly attenuated the DNA-triggered phosphorylation of IRF3 and TBK1 (Fig. 2d). These data suggest that RINCK is required for the DNA-induced activation of cGAS/STING signaling.

### RINCK is required for cGAMP synthesis

**Fig. 3:**
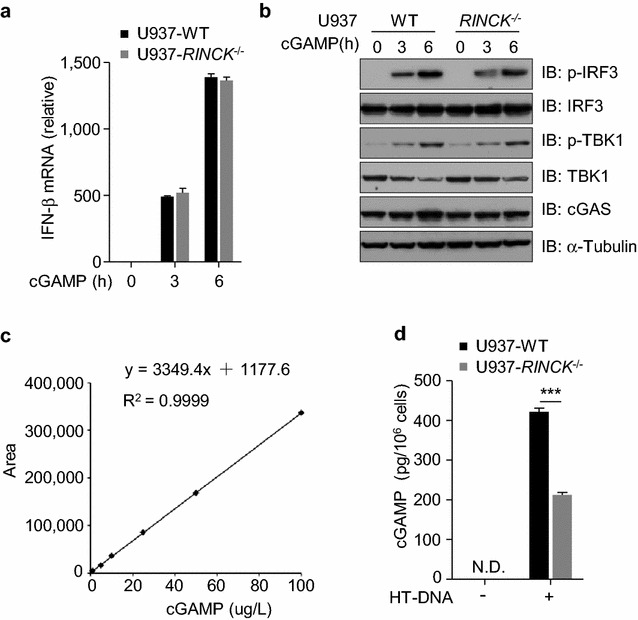
RINCK is required for cGAMP synthesis. **a** WT or RINCK-deficient U937 cells were treated with cGAMP for indicated time followed by measuring IFN-β mRNA with qPCR. **b** WT or RINCK-deficient U937 cells were treated with cGAMP for indicated time followed by immunoblotting with indicated antibodies. **c** Standard curve for cGAMP quantification by LC–MS/MRM. **d** WT or RINCK-deficient U937 cells were treated with HT-DNA for 6 h and the cell extract was collected for quantification of cGAMP by LC–MS/MRM. Data are presented as the mean ± SD. ***P < 0.001. N.D., not detected. Data represent three independent experiments

The engagement of cGAS with DNA leads to cGAS activation and the synthesis of cGAMP [5], which binds and activates the downstream adaptor protein STING and induces the phosphorylation of IRF3 and TBK1 [8]. To study how RINCK regulates cGAS/STING pathway, we used commercial cGAMP to treat both WT and RINCK-deficient cells. Our results show that cGAMP induced similar levels of the interferon production (Fig. 3a) and the phosphorylation of TBK1 and IRF3 (Fig. 3b). These data suggest that RINCK regulates cGAS/STING signaling at the cGAS level. We next assessed the whether RINCK regulates cGAS enzymatic activity. To do so, we established an approach to quantify cGAMP in cells by using the liquid chromatography–mass spectrometry/multiple reaction monitoring (LC–MS/MRM) (Fig. 3c). With this method, we measured the cGAMP synthesis in cells in response to HT-DNA treatment. We found that the production of cGAMP in RINCK-deficient cells was significantly decreased (Fig. 3d). Thus, RINCK appears to regulate the activity of cGAS directly.

### RINCK mediates the monoubiquitination of cGAS

**Fig. 4:**
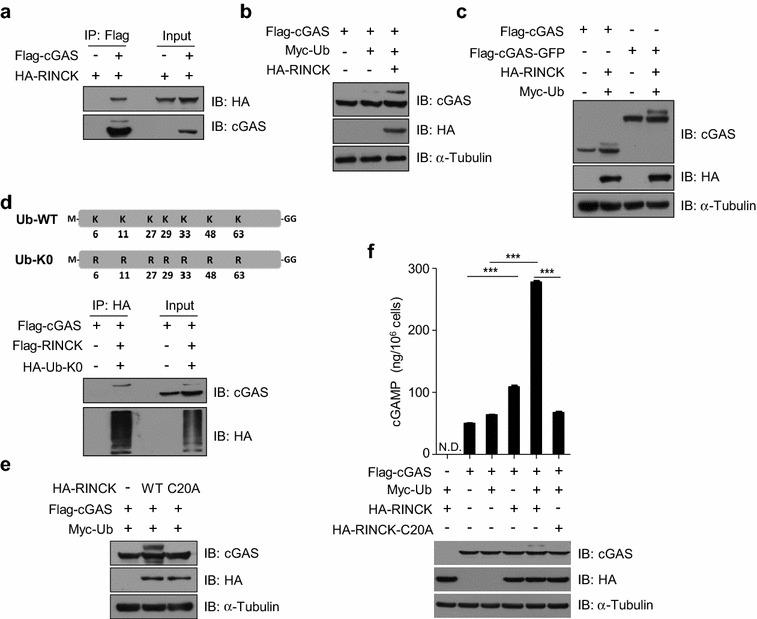
RINCK mediates the monoubiquitination of cGAS. **a** HEK293T cells were transfected with indicated plasmids for 24 h. Cell lysates were immunoprecipitated with anti-Flag antibody and then immunoblotted with indicated antibodies. **b**, **c** HEK293T cells were transfected with indicated plasmids for 24 h. Cell lysates were immunoblotted with indicated antibodies. **d** HEK293T cells were transfected with indicated plasmids for 24 h. Cell lysates were immunoprecipitated with anti-HA antibody and then immunoblotted with indicated antibodies. **e** HEK293T cells were transfected with indicated plasmids for 24 h. Cell lysates were immunoblotted with the indicated antibodies. **f** HEK293T cells were transfected with indicated plasmids for 24 h. Cell extract was collected for quantification of cGAMP by LC–MS/MRM. Data are presented as the mean ± SD. ***P < 0.001. N.D., not detected. Data represent three independent experiments

To further understand the mechanism by which RINCK regulates cGAS activation, we first examined the interaction of cGAS with RINCK. We transfected Flag-tagged human cGAS and HA-tagged RINCK into HEK293T cells and analyzed their interaction with immunoprecipitation. Consistent with our MS identification, the interaction of cGAS and RINCK was detected (Fig. 4a). Since RINCK is a known E3 ligase for ubiquitination [26], we then tested whether the presence of ubiquitin will enhance the interaction of cGAS and RINCK. Unexpectedly, when ubiquitin was co-transfected with cGAS and RINCK, we found that there was a band emerged above cGAS that can also be recognized by cGAS antibody (Fig. 4b). This result suggests that cGAS is likely modified by ubiquitin. By expressing the green fluorescence protein (GFP)-tagged cGAS with RINCK and ubiquitin, we confirmed this modification (Fig. 4c). Based on the shifted size of the modification band, we reasoned that the modification may be monoubiquitination [27]. To further confirm this, we used the K0 mutant of ubiquitin, in which all the seven Lysines were mutated to Arginin (R) and thus can only form monoubiquitination on target proteins [23]. With ubiquitin-K0, we confirmed that RINCK catalyzed the monoubiquitination of cGAS (Fig. 4d). Previous study showed that the C20A mutation blocks RINCK enzymatic activity [26]. Using RINCK^C20A^ mutant, we found that the mutated RINCK failed to mediate the monoubiquitination of cGAS (Fig. 4e). Together, these data suggest that RINCK mediates cGAS monoubiquitination. By co-expression of RINCK, ubiquitin and cGAS together, we found that WT RINCK, but not the RINCK^C20A^ mutant, dramatically increased cGAMP production in cells (Fig. 4f). Therefore, RINCK promotes cGAS activation by mediating the monoubiquitination of cGAS.

### RINCK promotes anti-DNA virus innate immune responses

**Fig. 5:**
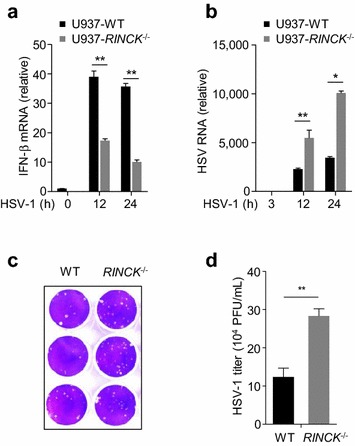
RINCK promotes anti-DNA virus innate immune responses. **a**, **b** WT or RINCK-deficient U937 cells were infected with HSV-1 (MOI = 1) for indicated time followed by measuring IFN-β mRNA and HSV-1 RNA with qPCR. **c** WT or RINCK-deficient U937 cells were infected with HSV-1 (MOI = 1) for 24 h. The replication of HSV-1 was measured by plaque assay in HeLa cells. **d** The viral titer (plaque-forming units, PFU) in **c** was calculated. Data are presented as the mean ± SD. *P < 0.05, **P < 0.01. Data represent three independent experiments

Since cGAS plays crucial role in anti-DNA virus immunity [28], we examined the role of RINCK in antiviral innate immune responses. We infected RINCK-deficient U937 cells with HSV-1, a DNA virus [29], and measured the interferon-β mRNA transcription by qPCR. Our results show that the interferon production was significantly reduced in *RINCK* knockout cells, compared to that in WT cells (Fig. 5a). We have also detected HSV-1 abundance by measuring the virus RNA transcription and found that HSV-1 RNA in RINCK-deficient cells was much higher than that in WT cells (Fig. 5b). Consistently, by performing the plaque assay, we found that RINCK deletion resulted in an increased HSV-1 virus load in cells (Fig. 5c, d). Taken together, these data indicate that RINCK is important for antiviral responses.

## Discussion

As a key DNA sensor, cGAS is crucial for immune defense against bacteria and virus infection [30]. Although cGAS has been shown to play a pivotal role in innate immunity, the exact regulation of cGAS activation is not fully understood. Our finding that RINCK positively regulates cGAS activation by mediating the monoubiquitination of cGAS uncovers the function of RINCK in cGAS-mediated innate immunity. The deficiency of RINCK resulted in dampened interferon production in response to cytosolic DNA or DNA virus infection. Recent studies showed that the deletion of cGAS fully rescued the self-DNA-induced autoimmunity [16], suggesting that inhibition of chronic activation of cGAS is critical for treating these diseases. On the other hand, effective activation of cGAS will also be important for antiviral immunity [28, 31]. Therefore, understand the detailed regulation of cGAS provide more targets for designing strategies to treat autoimmune diseases or viral infections.

Ubiquitination has been reported to play a pivotal role in modulating innate immune pathways, such as the retinoic acid-inducible gene I (RIG-I)-like receptor pathway, Toll-like receptor pathway, as well as the intracellular DNA sensing pathway [22, 32, 33]. Accumulating evidences suggested that more and more members of the tripartite motif (TRIM) family, which comprises about 70 proteins, are involved in pathogen-recognition and in host defenses [34, 35]. TRIM25 mediates the K63-linked ubiquitination of RIG-I and promotes the RIG-I-MAVS interaction to ensure the effective immune responses to RNA virus. The K63-linked ubiquitination of STING mediated by TRIM56 and TRIM32 promotes TBK1–STING interaction upon infection with Sendai virus (SeV) or HSV-1. RINCK is also a TRIM family member, TRIM41, localizes in both cytoplasm and nucleus, and is comprised of a RING, B-box and coiled-coil domain (CCD) [26]. It has been reported that RINCK regulates Protein Kinase C (PKC) signaling through ubiquitination [26]. Additionally, RINCK has also been shown to mediate the monoubiquitination of PKCε and links EGFR and NF-kB pathways in tumorigenesis [27]. Interestingly, a very recent publication reported that TRIM56 also mediates the monoubiquitination of cGAS [36]. Together with our study, monoubiquitination is likely to be an important regulation for cGAS activation.

Besides monoubiquitination, cGAS has been reported to be regulated by many other post-translational modifications, including phosphorylation, glutamylation, sumoylation, K27-linked and K48-linked polyubiquitinations [20, 21, 24, 25, 37, 38]. These studies, step by step, uncover the precise control of cGAS activation in cells. It remains to be further elucidated how these modifications work together to dynamically modulate cGAS activity. Since aberrant activation of cGAS is implicated in several autoimmune diseases, senescence and tumorigenesis [16, 17, 39], it will be important to develop means to modulate cGAS activation based on the regulatory mechanisms.

## Conclusions

By identifying that RINCK mediates the monoubiquitination of cGAS, our study uncovers a critical role of RINCK in the cGAS-mediated innate immunity.

## Methods

### Reagents

Anti-p-IRF3 antibody (4947) and anti-TBK1 antibody (3504) were purchased from Cell Signaling; anti-IRF3 antibody (ab68481) was from abcam; ISD was synthesized from Invitrogen; cGAMP (tlrl-cga23) was purchased from InvivoGen; anti-HA antibody (sc-7392) was purchased from Santa Cruz Biotechnology; anti-Flag M2 affinity gel (a2220), PMA (phorbol 12-myristate 13-acetate; 524400) and HT-DNA (D6898) were purchased from Sigma-Aldrich. Anti-cGAS antibody was prepared in our laboratory.

### Cell culture and transfection

U937 was cultured in RPMI-1640 medium containing 10% FBS, 2 mM l-glutamine, 100 U/ml penicillin and 100 mg/ml streptomycin. HEK293T, HeLa and Vero cells was cultured in Dulbecco’s modified Eagle’s medium (DMEM) containing 10% FBS, 2 mM l-glutamine, 100 U/ml penicillin and 100 mg/ml streptomycin. U937 cells were differentiated with PMA (0.1 μM) for 36 h before transfection or other treatment. HT-DNA and ISD treatment were performed with Lipofectamine 2000 (Invitrogen) at a final concentration of 2 μg/ml; cGAMP stimulation was performed as previously described [7, 40, 41], Briefly, cells were incubated with cGAMP (1 μg/ml) for 30 min at 37 °C in a permeabilization buffer [50 mM HEPES, pH 7.5; 100 mM KCl; 3 mM MgCl2; 0.1 mM DTT; 85 mM sucrose; 0.2% BSA; 1 mM ATP and 0.1 mM GTP; 1 μg/ml digitonin (Sigma, D141)]. The permeabilization buffer was replaced with RPMI-1640 medium and cells were further cultured for indicated time. siRNA transfection in HeLa cells was performed with Lipofectamine RNAiMAX (Invitrogen). RNA oligonucleotides for RINCK knockdown are as follows:

Rinck#1: 5′-ggcgagtgacagaactgaa-3′

RINCK#2: 5′-GACACGGTTTCTGGCTGAA-3′.

### Generation of CRISPR-Cas9 knockout cell line

The generation of RINCK deletion U937 cell line was performed as previously described [42]. The guide RNA sequence (Sense: 5′-CGGGGTCCGTGAAGTAATCG-3′; antisense: 5′-CGATTACTTCACGGACCCCG-3′) for human *RINCK* was designed by the online tool from Dr. Feng Zhang’s lab (http://crispr.mit.edu/).

### Plasmids

cDNA encoding RINCK, cGAS and Ubiquitin were sub-cloned into pCDNA3.0-Flag-vector, pXJ40-HA or pXJ40-Myc vector for expression in mammalian cells.

### Quantitative PCR (qPCR)

Total RNA was extracted with TRI reagent (93289, Sigma). qPCR was performed on an ABI StepOnePlus system according to manufacturer’s protocol. Data was analyzed with StepOnePlus software. Human *GAPDH* was used for normalization. The primers used to amplify the target genes are listed as follows:

**Table:** 

Gene	Forward primers (5′-3′)	Reverse primers (5′-3′)
Human *IFN*-*β*	AGGACAGGATGAACTTTGAC	TGATAGACATTAGCCAGGAG
Human *GAPDH*	GAGTCAACGGATTTGGTCGT	TTGATTTTGGAGGGATCTCG
Human *RINCK*	AGGAGGAGGAGGACGGAG	CTGGACCTGCTCATGCCACTG
Human *Rsad2*	TTGGACATTCTCGCTATCTCCT	AGTGCTTTGATCTGTTCCGTC
HSV-1 RNA	TGGGACACATGCCTTCTTGG	ACCCTTAGTCAGACTCTGTTACTTACCC

### Immunoprecipitation and immunoblotting

Cells were lysed with lysis buffer (20 mM Tris–HCl, pH 7.5; 0.5% Nonidet P-40; 250 mM NaCl; 3 mM EDTA and 3 mM EGTA) containing 20 mM *N*-Ethylmaleimide and complete protease inhibitor cocktail (Roche, 04693132001), followed by centrifugation at 20,000×*g* for 20 min at 4 °C. The supernatants were immunoprecipitated with anti-Flag M2 affinity beads. Cell lysates or immunoprecipitates were separated by SDS–PAGE and analyzed with immunoblotting. For the monoubiquitination detection, Cells were lysed with lysis buffer (20 mM Tris–HCl, pH 7.5; 0.5% Nonidet P-40; 250 mM NaCl; 3 mM EDTA and 3 mM EGTA) containing 20 mM *N*-Ethylmaleimide and complete protease inhibitor cocktail (Roche, 04693132001), followed by sonicating for 1 min and centrifugation at 20,000×*g* for 20 min at 4 °C. The supernatants were immunoprecipitated with anti-HA antibody and analyzed with immunoblotting.

### Elisa

PMA-differentiated U937 cells were seeded into 12-well plate at a density of 5 × 10^5^ cells/well and treated as indicated. The secreted interferon in cell culture medium was analyzed with ELISA kits (41410, PBL).

### cGAMP quantitative analysis

Cells were performed cGAMP extraction with extraction solvent [40:40:20 (v:v:v) methanol–acetonitrile–water] as described [43]. The quantification of cGAMP was performed on a triple-quadrupole mass spectrometer (Xevo TQ-S, Waters Corp. USA) equipped with an electrospray ionization source. The nebulizer gas was 99.95% nitrogen, and the collision gas was 99.99% argon with a pressure of 3 × 10E−3 mbar in the T-Wave cell. The gas flows of the cone and desolvation were set as 150 and 800 l/h, respectively. The target compound measurements were performed in the positive mode with a 3.5 kV capillary voltage, 120 °C source temperature, and 450 °C desolvation temperature. The optimized ion transitions were: cGAMP m/z 675 → 524; m/z 675 → 136.

### Statistical analysis

No statistical methods were used to estimate sample size. A standard two-tailed unpaired Student’s *t* test was used for statistical analysis of two groups. Statistical analyzed data are expressed as mean ± SD. *P* value < 0.05 is considered as statistically significant. We performed the statistical analysis using GraphPad Prism.

## Abbreviations

cGAMP: cyclic GMP-AMP
cGAS: cyclic GMP-AMP synthase
RINCK: RING finger protein that interacts with C kinase
TRIM: tripartite motif protein
CRISPR/Cas9: clustered regularly interspaced short palindromic repeats and the endonuclease Cas9
HSV-1: herpes simplex virus 1
TBK1: TANK-binding kinase 1
IRF3: interferon regulatory factor 3
